# Is There a (Host) Genetic Predisposition to Progressive Multifocal Leukoencephalopathy?

**DOI:** 10.3389/fimmu.2015.00216

**Published:** 2015-05-11

**Authors:** Eli Hatchwell

**Affiliations:** ^1^Population Diagnostics UK, Inc., Begbroke Science Park, Begbroke, Oxfordshire, UK

**Keywords:** progressive multifocal leukoencephalopathy, host genetic factors, primary immunodeficiencies, PML predisposition, natalizumab

## Abstract

Progressive multifocal leukoencephalopathy (PML) has been described in association with a variety of predisposing risk factors, including HIV/AIDS, lymphoproliferative disorders and, most recently, treatment with a range of biologics, most notably natalizumab in multiple sclerosis (MS) (1). However, while these underlying disorders appear to be (usually) necessary, they are not sufficient to predict the development of PML, since only a small fraction of such individuals will succumb, raising the question of whether host genetic factors must also play a role. Evidence is mounting that this is, indeed, the case but more work needs to be done to fully delineate these underlying genetic factors. Finally, while it is possible that an underlying genetic susceptibility for PML *in general* will be uncovered in the future, the current evidence argues for a collection of multiple, individually rare underlying susceptibilities (with one predominant single genetic susceptibility in each individual), as described in this review.

## Introduction

Progressive multifocal leukoencephalopathy (PML) is a severe, potentially fatal, CNS infection. It is “caused” by reactivation of the JC virus (JCV). However, while JCV is present at very high rates in the general population, PML remains a rare disorder, albeit an important one because of the clinical sequelae, and the recently demonstrated association with a variety of useful therapies, in particular natalizumab in multiple sclerosis (MS). A number of risk factors for PML have been described but these are better viewed as necessary but *not* sufficient. The majority of individuals with these risk factors do *not* develop PML and so the obvious question is *“In addition to the well-known risk factors for PML, are there host genetic factors that also play a role?”*

## “Traditional” Risk Factors for PML

This is a topic that has been well reviewed in the literature [for a recent comprehensive discussion, see Ref. (1)]. In brief, while rare individuals have been reported, in whom no obvious risk factors are present (2), the majority of individuals have a history of profound cellular immunosuppression, whether associated with systemic disorders, such as HIV, lymphoproliferative disease, rheumatological disorders (SLE, for example), or else treatment with immunosuppressive or immunomodulatory therapies. Historically, PML (first described in the 1950s) was known mainly as a disease associated with lymphoproliferative disorders. Knowledge of PML increased with the advent of AIDS, as 4–5% of patients developed PML. In recent years, PML has come to prominence in the context of the drug natalizumab, an effective biologic used in the treatment of MS. PML has also been described in association with other biologics [reviewed in Ref. (1)] and, most recently, with the use of drugs containing fumarates (used in the treatment of psoriasis and also the MS drug Tecfidera) (3).

The important point is that while these risk factors are highly relevant, they do not, on their own, predict who will develop PML, since the vast majority of individuals with these risk factors will not develop the disorder. Clearly, other factors need to be considered and there is growing evidence for the role of host genetic factors in susceptibility to PML.

Finally, the rare description of individuals that have developed PML in the complete absence of any known “traditional” risk factor argues very strongly for the presence of host genetic factors that result in an inability to effectively deal with JCV.

## Precedents for Genetic Predisposition to Specific Infectious Disease

Many factors/conditions are known to predispose to infectious disease in general. However, what is of greater interest is the existence of genetic syndromes that appear to predispose to severe infection with very *specific* infectious agents. A classical example is X-linked lymphoproliferative syndrome (XLP), a primary immunodeficiency disorder (PID) characterized by severe immune dysregulation often after viral infection, typically with Epstein–Barr virus (EBV). XLP is present in two forms, each associated with mutations in a different gene: XLP1 is associated with SH2D1A mutations, while XLP2 is associated with XIAP mutations. Both genes are located in Xq25 (4). More recently, Herpes simplex virus encephalitis (HSE), a rare complication of infection with HSV-1 has been shown to be associated with deficiency of TLR3 at 4q35.1 (5).

## Known/Suspected Genetic Predispositions for PML

The association of PML with rare immune deficiency syndromes is suggested by a number of case reports, some of which predated the ability to confirm the clinical diagnosis of the underlying disorder by specific gene testing.

Table 1 summarizes specific PIDs in which PML has been reported, along with estimates for the numbers of distinct PML cases observed.

**Table 1:** **Summary of specific PIDs reported in association with PML**.

Disorder predisposing to PML	Gene	Number PML cases	Reference
Wiskott–Aldrich Syndrome	WAS, WIPF11	5	(6–10)
Severe combined immunodeficiency syndrome	Various	2	(11, 12)
X-linked hyper-IgM Syndrome	CD40LG	3	(13–15)
Hyper IgE Syndrome	DOCK8, STAT3, TYK2, STK3	2	(16, 17)
ICF syndrome	DNMT3B	1	(18)
Adenosine deaminase deficiency	ADA	1	(19)
Idiopathic CD4 deficiency	RAG1, MST1, LCK, ITK	1	(20)
X-linked agammaglobulinemia	BTK	1	(21)
Purine nucleoside phosphorylase deficiency	PNP	1	(22)
STAT1 gain of function	STAT1	1	(23)

*Note that references refer to PML case reports but genes listed refer to known causes of the syndrome, even if some specific genes have not yet been implicated in PML *per se**.

### Wiskott–aldrich syndrome

Wiskott–Aldrich syndrome (WAS) is an X-linked recessive immunodeficiency, caused by mutations in the WAS gene at Xp11.23. A similar disorder, WAS2, is caused by recessive mutations in WIPF11, at 2q31.1. There are five reports of PML in association with clinically diagnosed WAS. It is unclear why WAS is the most common PID described in association with PML. This may simply be due to ascertainment bias, given that the small number of cases reported early on may have alerted clinicians to specifically test for this condition. Alternatively, this may simply be a sampling issue, given the rarity of PML.

### Severe combined immunodeficiency syndrome

Severe combined immunodeficiency syndrome (SCID) refers to a genetically and clinically heterogeneous group of disorders with defective cellular and humoral immune function. There have been at least two reports of PML in association with SCID.

### X-linked hyper-IgM syndrome

X-Linked Hyper-IgM Syndrome is caused by mutations in the CD40LG (aka TNFSF5) gene at Xq26.3. It is a rare immunodeficiency characterized by normal/elevated IgM levels but markedly decreased IgG, IgA, and IgE resulting in a profound susceptibility to bacterial infections and an increased susceptibility to opportunistic infections. It has been described in four patients reported as having PML.

### Hyper-IgE syndrome

Hyper-IgE recurrent infection syndrome is a PID characterized by chronic eczema, recurrent staphylococcal infections, increased serum IgE, and eosinophilia. It may be associated with dominant mutations in STAT3 at 17q21.2, recessive mutations in DOCK8 at 9p24.3, or recessive mutations in TYK2 at 19p13.2 (only one case reported). Most recently, a patient with PML was reported with a >500 kb homozygous deletion encompassing a number of genes in 9p24.3, with DOCK8 the strongest candidate for PML susceptibility. Finally, there was also a report of a patient with PML associated with DOCK8 mutation in 2010.

### ICF syndrome

Immunodeficiency, Centromeric instability, and Facial dysmorphism (ICF) syndrome is a rare autosomal recessive syndrome associated with mutations in DNMT3B at 20q11.21. One PML case has been reported in a patient with ICF.

### Adenosine deaminase deficiency

Inherited Adenosine Deaminase (ADA) deficiency causes a spectrum of disease, from SCID, presenting in infancy and usually resulting in early death, to a later onset at age 6–24 months, and, finally, a small percentage of cases with a “later” onset, presenting from 4 years of age to adulthood. The disorder is associated with mutations in ADA at 20q13.12. There has been one case of PML reported in the context of ADA deficiency.

### Idiopathic CD4 deficiency

Idiopathic CD4 lymphocytopenia (ICL) is a syndrome described in patients with low counts of CD4 cells and no other causes for immunosuppression. A few cases of PML have been described in association with this entity. Mutations in a number of genes have been reported in association with ICL, including RAG1 at 11p.12, MST1 at 3p21.31, LCK at 1p35.1, and ITK at 5q33.3. One case of PML has been reported in association with ICL.

### X-linked agammaglobulinemia

X-linked agammaglobulinemia (XLA) is caused by mutations in the BTK gene at Xq22.1. It results in an immunodeficiency characterized by failure to produce mature B lymphocytes and is associated with a failure of Ig heavy chain rearrangement. One case of PML has been reported in association with XLA.

### Purine nucleoside phosphorylase deficiency

Purine nucleoside phosphorylase (PNP) deficiency is a rare autosomal recessive immunodeficiency disorder characterized mainly by decreased T cell function. It is caused by mutations in the PNP gene at 14q11.2 One case has been reported with PML.

### STAT1 gain of function

Mutations in STAT1 (2q32.2) have been implicated in a number of disorders, including susceptibility to a variety of infectious agents, depending on which of four main subtypes of STAT1 is present. In one study describing the association between STAT gain of function mutations and disseminated coccidioidomycosis and histoplasmosis, one of five patients was reported with PML.

## Challenges of Genetic Studies into PML

While the reports of rare individuals with PML in association with a confirmed PID immediately suggest a set of candidate genes that should be routinely searched for in PML patients, even when no such obvious (clinical) diagnosis has been made, it is possible that a more subtle and specific immunodeficiency will explain the majority of cases of PML, namely an immunodeficiency that is *not* clinically recognizable except in the context of JCV (i.e., one that does not result in other opportunistic infections, as is the case for XLP and specific susceptibility to EBV, or has other obvious associated clinical features that would lead to a diagnosis of a known PID). However, it is important to note that precedents *do* exist for the role of Mendelian genes, initially identified in severe disorders, being relevant to general populations in whom the severe phenotype is not present. An example of this has recently been demonstrated in late-onset Alzheimer’s disease (LOAD), with the finding that rare variants in PSEN1, PSEN2, and APP increase the risk of Alzheimer’s in LOAD families.

While a candidate gene list presents itself, there do remain significant challenges for the genetic dissection of PML predisposition, not only because the examples of PIDs described in association with PML are likely to account for only a small subset of the overall susceptibility to this disorder but because of more general considerations.

Progressive multifocal leukoencephalopathy remains an uncommon disorder, whose geographical distribution is wide. Very few specialists/centers have seen more than a handful of cases and collecting samples from sufficient numbers of cases has been challenging. Furthermore, based on the number of distinct PIDs in which PML has been reported in at least some cases, it is likely that the number of genes that might predispose to PML is significant, so that the unbiased identification of rare, causal variants will prove very difficult using modern whole genome or exome sequencing methods, for example, since the degree of background variation is simply too great to achieve statistical significance. The challenge of using next generation sequencing approaches is exacerbated by the possibility that some mutations *may* be dominantly acting (i.e., heterozygotes have the genetic susceptibility), a class of variants for which inferring causality is much more challenging than recessive disorders. Furthermore, the usual “tricks” employed to find significant causal variants in human genetic disorders either via traditional approaches or using NGS cannot be used because:

There are no:

1. families with Mendelian inheritance of PML (useful for traditional linkage analysis and/or segregation analysis of variants in suspected candidate genes), since, even were a dominant mutation to be segregating, it is highly unlikely that all carriers would also have the other, requisite, predispositions (underlying cellular immune suppression of whatever cause, for example);
2. obvious examples of PML arising as a result of a *de novo* mutation [in some childhood syndromes, such as severe intellectual disability (where parents are phenotypically normal), searches for de novo mutations have been fruitful];
3. other clinical/phenotypic clues that may aid in delineation of more specific subsets, for which correlations with underlying genetics may be useful (as occurs frequently in ID, wherein many ID syndromes are associated with specific physical manifestations that permit more specific disease delineation).

Discovery of the set of variants that underlie predisposition to PML is likely to depend on a combination of

1. consideration of strong candidate genes, in addition to those linked to already described PIDs. However, this is still a large class, based on the high number of genetically distinct conditions that are associated with immune suppression;
2. unbiased, genome-wide searches for causal variants, coupled with intelligent filtering of variants, based on known PIDs along with gene function as related to the immune system. However, such genome-wide searches for novel variants underlying PML development will not be straightforward;
3. the use of larger cohorts of PML subjects than has hitherto been possible.

## Potential Benefits of Uncovering Genetic PML-Susceptibility Variants

In the short term, the ability to more accurately predict who is at high risk of developing PML will be of enormous benefit in the context of drug treatment with compounds (mostly biologics) that are highly effective in their disease context (natalizumab in MS, for example) but carry a small risk of a devastating disorder. The hope is that patients in whom a course of treatment is being planned with such a drug would undergo initial, companion diagnostic testing, in order to effectively exclude those that were at high risk of PML, in the process reassuring those with negative tests about their dramatically reduced risk of developing PML.

Currently, the only approach in this direction is based on testing for the presence of JCV, since JCV is an absolute pre-requisite for PML development (24). However, because JCV is present in a large percentage of the normal population, this approach effectively excludes a significant proportion of patients from a treatment that is highly effective. In fact, it is possible that testing for JCV virus will not achieve its stated aim and patients will elect to accept the risk of PML, in return for improved quality of life on drug treatment (e.g., natalizumab in MS).

## Conclusion

There appear to be three major criteria that must be fulfilled before PML occurs:

1. JCV is present;
2. some form of cellular immune suppression is present, whether due to underlying systemic disease (such as hematological malignancy) or treatment with immunosuppressives (especially biologics, such as natalizumab);
3. host (genetic) susceptibility to develop PML;

For those rare individuals in whom no specific immune suppression is present in the medical history, there are two probably only essential factors:

1. JCV is present;
2. host (genetic) susceptibility to develop PML.

While there has been good progress in unraveling the epidemiology of PML, there is now some urgency to uncover as many of the underlying genetic variants as possible, primarily to create an effective and specific screening test that will determine, as definitively as possible, which patients should not be treated with a given therapeutic (MS, for example, when natalizumab is being considered) and which patients can safely (with dramatically reduced risk) be treated.

Finally, an improved understanding of the etiology of PML may, in the longer term, provide insights that will lead to therapies for individuals in whom PML has arisen as a result of their underlying disease, rather than a chosen treatment (e.g., HIV/AIDS and lymphoproliferative disorders).

## Conflict of Interest Statement

Eli Hatchwell is an employee of Population Diagnostics, Inc., UK.
